# Methane emission, nitrogen excretion, and energy partitioning in Hanwoo steers fed a typical TMR diet supplemented with *Pharbitis nil* seeds

**DOI:** 10.3389/fvets.2024.1467077

**Published:** 2024-09-24

**Authors:** Rajaraman Bharanidharan, Panyavong Xaysana, Woo Hyeong Hong, Taehoon Kim, Jun Suk Byun, Yookyung Lee, Byamungu Mayange Tomple, Kyoung Hoon Kim, Ridha Ibidhi

**Affiliations:** ^1^Department of Eco-friendly Livestock Science, Institute of Green Bio Science and Technology, Seoul National University, Pyeongchang, Republic of Korea; ^2^Graduate School of International Agricultural Technology, Seoul National University, Pyeongchang, Republic of Korea; ^3^Department of Geography, McGill University, Montreal, QC, Canada; ^4^University of Maryland Center for Environmental Science, Frostburg, MD, United States; ^5^National Institute of Animal Sciences, Rural Development Administration, Cheonan, Republic of Korea; ^6^National Institute of Animal Sciences, Rural Development Administration, Hamyang, Republic of Korea; ^7^Agroécologie, INRAE, Institut Agro, Université de Bourgogne, Université Bourgogne Franche-Comté, Dijon, France

**Keywords:** energy partitioning, Hanwoo, methane, nitrogen utilization, *Pharbitis nil*

## Abstract

Two *in vivo* experiments were conducted to evaluate the potential of *Pharbitis nil* seeds (PA) as an anti-methanogenic additive to ruminant feed. In experiment 1, six Hanwoo steers (459.0 ± 25.8 kg) were fed either a total mixed ration (TMR; 32-d period) or TMR supplemented with PA at 5% dry matter (DM) intake (TMR-PA; 45-d period) for two consecutive periods. Fecal and urine outputs were measured in an apparent digestibility trial in both periods. Methane (CH_4_) yield and heat energy (HE) were measured using respiratory chambers equipped with gas analyzers. In experiment 2, five rumen cannulated Holstein steers (744 ± 35 kg) were fed the same TMR or TMR-PA diets for 40 days; rumen samples were collected at 0, 1.5, and 3 h after feeding on the last day of the feeding period. In experiment 1, although there were no differences (*p* > 0.05) in nutrients or gross energy intake (GEI) between the groups, an increase (*p* < 0.05) in the apparent digestibility of DM (9.1%) and neutral detergent fiber (22.9%) was observed in the TMR-PA fed Hanwoo steers. Pronounced decreases (*p* < 0.05) in CH_4_ (g/Kg DM; 17.1%) and urinary N excretion (% N intake; 7.6%) were observed in the TMR-PA group, leading to a 14.7% increase in metabolizable energy intake (% GEI). However, only a numerical increase (*p* > 0.05) in retained energy was observed due to the increase in HE loss. In experiment 2, a drastic decrease (*p* < 0.05) in rumen ammonia concentration (56.3%) associated with an increased (*p* = 0.091) rumen short-chain fatty acid concentration 1.5 h after feeding were observed in TMR-PA fed Holstein steers. A 26.6% increase (*p* < 0.05) in the propionate proportion during the treatment period clearly reflected a shift in the ruminal H_2_ sink after 3 h of feeding. A 40% reduction (*p* = 0.067) in the relative abundance of rumen protozoa *Entodinium caudatum* was also observed. It was concluded that PA could be a natural feed additive for CH_4_ and N emission abatement.

## 1 Introduction

In addition to their environmental footprints, methane (CH_4_) and nitrogen (N) emissions represent losses in livestock dietary energy and N utilization (1, 2). The total farm-gate greenhouse gas emissions from Korea were 19.3 MtCO_2_ eq/year in 2021; CH_4_ and nitrous oxide (N_2_O) from livestock farming (enteric fermentation and manure management) contributed 6.59 and 1.05 MtCO_2_ eq/year, respectively (3). The recent update of the nationally determined contribution of Korea includes a target to reduce the total absolute greenhouse gas emissions by 24.4% from their 2017 level (709.1 MtCO_2eq_; i.e. reducing national greenhouse gas emissions to 536 MtCO_2eq_) by the year 2030 (4). Furthermore, Korea has pledged to become carbon neutral by 2050 with a focus on low-carbon agricultural technologies targeting the development of low-methane feeds that improve livestock enteric fermentation and reduce CH_4_ emissions (5). However, the substantial increase in the total cattle population, with a record of 3.35 million heads in the first quarter of 2024 (6), has raised concerns regarding their associated CH_4_ and N_2_O emissions.

Several dietary interventions have been proposed to mitigate the emissions of CH_4_ and noxious N-containing compounds from ruminant husbandry (7, 8). The implementation of these interventions while avoiding changes to nutrient and energy utilization is a major goal in animal nutrition. Large-scale identification of plant materials rich in plant secondary metabolites, such as tannins and saponins, for CH_4_ and N abatement in the livestock industry has been achieved in recent years (9, 10). Plant materials rich in bioactive lipid compounds and other unsaturated fatty acids (FAs) have been extensively studied to elucidate these effects (7, 8). However, most relevant results have been obtained by *in vitro* trials; only few plant materials such as hazel leaves (11, 12), tannin rich legumes (13, 14), and conventional or unconventional oil seeds (15–17) have been studied *in vivo* to determine their nutritional value and effects on CH_4_ production and N utilization. Moreover, inconsistent results (18, 19) and adverse effects on nutrient digestibility (20) have encouraged ruminant nutritionists to explore novel feed additives with high nutritional value.

*Pharbitis nil* (Convolvulaceae) is an annual climbing herb widely distributed throughout Korea, Japan, and China, and are reported to be enriched in resin glycosides (21), chlorogenic acid derivatives (22), and other phenolic substances (23). Seeds of *Pharbitis nil* (PA) were also used as a purgative agent and in treating digestive disorders (24). Our previous *in vitro* and *in sacco* studies identified and evaluated the potential of PA for mitigating ruminal CH_4_ and NH_3_ production with improved nutrient digestibility at a range of 4.5%−9% dry matter (DM) supplementation (10, 25). We also found that PA is rich in nutrients such as neutral detergent fiber (NDF), crude protein (CP), and fat (25). Our previous studies demonstrated that the abundance of flavonoids and unsaturated FAs in PA probably had detrimental effects on ciliate protozoa (25), which are generally regarded as H_2_ producers and are detrimental for ruminal N metabolism (8, 26). Therefore, the inclusion of PA in ruminant diets would be an effective strategy to prevent inefficient N utilization and CH_4_ emission. However, *in vivo* experiments may be more appropriate for evaluating the nutritive value of PA because a drastic change in the Bacteroides: Firmicutes ratio was observed after PA incubation in rumen due to a probable defaunation effect (25).

Therefore, the present study investigated the effects of supplementing PA in the diet of Hanwoo steers at 5% DM intake on digestibility, CH_4_ emissions, energy and N balance, and rumen protozoal diversity via reverse transcription quantitative polymerase chain reaction (RT-qPCR) analysis. We hypothesized that PA supplementation to ruminants would enhance nutrient digestibility and decrease protozoal abundance, followed by reductions in N and CH_4_ emissions, thereby increasing dietary metabolizability and allowing spare energy to be used for productivity gains.

## 2 Materials and methods

### 2.1 Animal ethics statement

All experimental animals were obtained from the animal farm of Seoul National University. The methods and protocols for all experiments involving animals were approved by the Committee for the Institutional Animal Care and Use of Seoul National University (SNU-210615-3), and all experiments were performed in accordance with relevant guidelines and regulations.

### 2.2 Experiment 1: effects of PA on CH_4_ production, energy balance, and productivity of Hanwoo steers

In experiment 1, six Hanwoo steers with an initial body weight (BW) of 459.0 ± 25.8 kg were randomly allocated to pens equipped with automatic Calan doors. All steers were offered a commercial total mixed ration (TMR; Table 1) as a basal diet for 21 days and allowed to adapt to the Calan doors to consume their individual feeds prior to commencement of experimental work. The experiment included two successive periods where the animals were first fed a basal diet for 32 days (TMR), followed by 45 days of PA supplementation at 5% DM (TMR-PA) of the basal diet. The TMR period included 21 days for diet adaptation, 3 days for respiratory chamber adaptation, 3 days for gas sampling, and 4 days for total feces and urine collection. The TMR-PA period included 35 days for diet adaptation, 3 days for respiratory chamber adaptation, 3 days for gas sampling, and 4 days for total feces and urine collection. Because only three steers could be housed in the respiratory chambers at a given time, steers began the experiment in groups of three, staggered by 1 week. The TMR diet was prepared every 14 days during the experimental period by a local feed mill. The prepared TMR diet was then packed in vinyl bags (20 kg per bag) and stored for feeding to the animals. The diet was provided at 2% initial BW twice daily (at 09:00 and 18:00), and the animals had unrestricted access to water. PA was purchased from the local market in Dongdaemun-gu, Seoul, Republic of Korea, ground to pass through a 1-mm screen (Model 4, Thomas Scientific, Chadds Ford Township, PA, USA), and mixed with the diet during feeding. The BWs of the animals were measured both at the beginning and end of the period to calculate the average daily gain (ADG). Feed refusals were recorded both in the pen and respiratory chambers to determine the DM intake and feed conversion ratio. Samples of feed and PA were collected at the beginning of each period, whereas samples of refusals were collected each day when the animals were in respiratory chambers. All samples were stored at −20°C until further analysis.

**Table 1:** Ingredients and chemical compositions of the dietary treatments.

	**TMR**	**TMR-PA**
**Ingredient composition, %DM**		
Corn DDGS	17.5	16.6
Corn gluten feed	11.0	10.4
Rye straw	10.7	10.2
Steam-flaked corn	9.7	9.2
Broken corn	9.4	8.9
Wheat flour	8.8	8.5
Fermented mixture^a^	7.3	7.0
Palm Kernel meal	7.0	6.6
Whole cottonseed	5.0	4.7
Rice bran	3.0	2.8
Tall fescue straw	2.6	2.5
Wheat bran	2.0	1.9
Pineapple pulp meal	0.6	0.5
Sodium bicarbonate	0.3	0.3
Limestone	0.1	0.1
*Pharbitis nil* seeds	–	5.0
**Chemical composition, %DM**		
Organic matter (OM)	93.3	93.3
Crude protein (CP)	16.4	16.7
Ether extract (EE)	4.8	5.2
Neutral detergent fiber (NDFom)^b^	38.7	38.9
Acid detergent fiber (ADFom)^c^	18.5	18.4
Gross energy (GE, MCal/kg)	4.3	4.4

TMR, total mixed ration; TMR-PA, TMR supplemented with Pharbitis nil seeds at 5% DM.

^a^Rice bran 50% and wheat bran 50% (w/w) with 0.1% Lactobacillus acidophilus KCTC3140 (1.2 × 10^7^ CFU/ml) and 0.1% Saccharomyces cerevisiae KACC30068 (2.1 × 10^6^ CFU/ml) were mixed and maintained at the optimal temperature for fermentation.

^b^Neutral detergent fiber assayed with heat-stable amylase and expressed exclusive of residual ash.

^c^Acid detergent fiber expressed excluding residual ash.

#### 2.2.1 Respiratory chamber description and gas sampling for methane and heat production measurements

Gas sampling was performed for three consecutive days, beginning from days 27 and 40 during the TMR and TMR-PA periods, respectively. Animals were placed in the chamber; the CH_4_, CO_2_, and oxygen (O_2_) concentrations were measured using three indirect open-circuit whole-body respiratory chambers composed of steel frames and covered by detachable, clear polyvinyl chloride (PVC) sheets to enable visual communication between animals. The design and operating procedures of the respiratory chambers were developed based on those previously reported in Cesar and Garry (27). The outer dimensions of each chamber were 137 (width) × 356 (depth) × 200 (height) cm, resulting in a chamber volume of 9.7 m^3^. Each chamber was equipped with a feeder and water bowl; the chamber floor was layered with a rubber mat containing a metal grated opening (80 × 80 cm) in the middle. Each chamber was fixed to the floor; the gap between the floor and the bottom of the chamber (36 cm) was sealed by covering the middle opening with a movable steel container for urine collection. All chambers were designed to maintain ambient temperature and relative humidity at 21°C and 60%, respectively, using an independent regulation system that contained an air refrigeration unit (model DK-C-150E; Dryer Korea) with a recirculating fan (model ALFFIZ-WBCAI-015H; Busung, Korea) and an air filter section. This unit was integrated into the chamber through 10-cm-diameter flexible thermo-insulating tubes from the ceiling. For recirculation, air left the chamber (0.6 m/s) through openings in the middle of the chamber ceiling; the dehumidified air was then recycled into the chamber (0.1 m/s) through openings at the back end of the chamber ceiling. The speed of air circulation was fixed to avoid loss of gases and maximize their recovery. Condensed water from the refrigeration unit was drained into the chamber through a capillary tube attached to the water traps.

Streams of ambient air from the barn were drawn through a 10-cm-diameter opening in the back door of each chamber by a sealed rotary pump connected to a flow meter (model LS-3D; Teledyne Technologies, Thousand Oaks, CA, USA) that provided a constant wet ventilation rate (wet VR) of 450 L/min. Each chamber was fitted with an air outlet containing a filter box in the front section of the ceiling, and the air was continuously drawn out next to the control room through a 10-cm-diameter polyvinyl chloride (PVC) pipe. Air outflow from each chamber was sampled from the middle of a PVC pipe between the chamber and the exhaust pump, through a gas tube connected to the analysis system.

All three chambers shared a common gas analysis system that consisted of a multiplexer (Metabolic Controller, B.S. Technolab, Seoul, Korea) connected to a gas sampling pump (Columbus Instruments, Columbus, OH, USA) and a gas analyzer (model VA-3000, Horiba Ltd., Tokyo, Japan) containing non-dispersive infrared CH_4_ (0–2,000 ppm) and CO_2_ (0–10,000 ppm) gas sensors, as well as a paramagnetic O_2_ (0–25%) sensor. The multiplexer had a gas switching system with eight measuring channels that controlled the three chambers and background air samples; it delivered only one sample stream (from one of the chambers or background) to the analyzer set through a sample flow that was set to 0.6 L/min by the pump. The sample used to determine background gas concentrations was acquired near the inlet of each chamber. Before each air sample entered the analysis system, it was filtered and dried in cylindrical plastic columns filled with desiccants and an indicator (Mesh size 8, Drierite, Xenia, OH, USA). The sample stream was then sequentially delivered to the CH_4_, CO_2_, and O_2_ analyzers. Calibrations of gas sensors (zero and span) were performed before the beginning of each measurement using standard gas mixtures with concentrations of 807 μmol/mol CH_4_, 7,996 μmol/mol CO_2_, and 19.99 %mol/mol O_2_ in N (Air Korea, Seoul, Korea).

Data acquisition and analysis software (Metabolic System, SciTech, Seoul, Korea) was used to record the CH_4_, CO_2_, and O_2_ concentrations, as well as the wet VR, during the measurement period. The time interval of multiplexer channel switching was also defined by the operator in the data acquisition software. Based on the stabilities of gas concentrations and numbers of operating chambers, the air was sampled every 10.5 min for each chamber, with background air and chamber air sampling sequences of 90 and 120 s, respectively. The average value of the final 20 s of sampling time was considered for calculations.

Before animal placement in respiratory chambers, the recovery rate of each chamber was tested in each period using a standard CH_4_ gas mixture (25% mol/mol balance N_2_; Air Korea). Briefly, a fixed volume of CH_4_ (50 ml/min) was injected into each chamber through a gas tube connected to a portable mass flow meter. The gas tube was placed near the feeder to accurately simulate animal emissions during feeding. The gas was allowed to mix for 10 min to achieve an equilibrium state with the inlet and outlet air passages closed. After 10 min, the inlet and outlet air passages were opened; inlet and outlet gases were sampled as described above. The difference in CH_4_ concentration between outlet and inlet gases (DCH_4__ppm_), known ventilation rate (wet VR), and the known injected CH_4_ concentration (InCH_4ml/min_) were used to calculate the gas recovery rates of the chamber using the following formula:


$$\begin{array}{r} Theoretical\;CH_{4ppm}\left(ThCH_{4ppm}\right)=\left(InCH_{4mL/min}/wetVR_{mL/min}\right)\\ \times 1,000,000\\ Gas\;recovery\;rate\;\left(\%\right)=\left(DCH_{4\;ppm}/ThCH_{4\;ppm}\right)\times 100 \end{array}$$

During the experiment, the same routine operations were conducted when animals were placed in the chamber, and CH_4_ emission was calculated using the formula:


$$\begin{array}{r} CH_{4}\;emission\left(L/min\right)=(wetVR_{L/min}\times \\ (\left[DC{H_{4}}_{ppm}\right]/1,000,000))/gas\;recovery\;rate(\%) \end{array}$$

The same formula and gas recovery rate (%) were used to determine CO_2_ emissions by substituting the values of DCH_4__ppm_ with DCO_2__ppm_. For the determination of O_2_ consumption, the oxygen DO_2%_ observed during the experiment was normalized and scaled up with the DO_2%_ observed during the recovery test.

#### 2.2.2 Digestibility and N balance trial

Feces and urine collection were performed for four consecutive days beginning on days 29 and 42 of the TMR and TMR-PA periods, respectively, immediately after gas measurements. For collection of total feces and urine, clear PVC sheets were detached from the chamber and the chamber itself was used as a metabolic crate. The animals were housed on rubber mattresses and allowed free access to feed and fresh drinking water. The feces voided daily from each animal on the rubber mat were collected using a shovel and dragged into a stainless-steel box located at the end of each metabolic crate. The daily total feces output was collected at 09:00 each day and recorded. The feces were then homogenized; 10% of the total feces from each animal was sampled and stored at −20°C. The total urine excreted from each animal was also collected daily using a custom-made pyramidal silicone funnel attached to the animal's lower body. The funnel was connected to a rotary pump and the excreted urine drained directly into clean 25-L airtight plastic containers via polypropylene tubing. In each container, 300 ml of 4N H_2_SO_4_ were included daily to acidify urine upon entry. The daily total urine output was recorded and homogenized; 10% of the urine volume from each animal was sampled and stored at −20°C. All daily subsamples were mixed; 10% of the total mixed sample was retained and used for further analysis.

#### 2.2.3 Chemical analyses

Feed, refusals, PA, and feces were dried in a forced-air oven at 65°C for 72 h, then ground to pass through a 1-mm screen (Thomas Scientific Model 4, NJ, USA). The ground samples were subjected to DM analysis by drying at 105°C for 3 h. The OM was determined after ashing at 600°C using a Nabertherm LE 14/11/R7 Compact Muffle Furnace (Bahnhofstr, Lilienthal, Germany) for 3 h (28). Ether extract (EE) was determined using an ANKOM^XT15^ Extractor (ANKOM Technology Corp., Fairport, NY, USA) after a filter bag procedure (29) with petroleum ether as the solvent. NDF and acid detergent fiber were measured using the ANKOM A2000 fiber analyzer (ANKOM Technology Corp.) filter bag technique. NDF content was determined with a heat-stable amylase and expressed exclusive of residual ash (amylase neutral detergent fiber organic matter [aNDFom] (30). Acid detergent fiber was measured using the method of Van Soest (31), and the results were presented excluding residual ash (aNDFom). The N contents of feeds, refusals, PA, feces, and acidified urine were determined using the Kjeldahl method (Kjeltec Auto Sampler System, 8400 Analyzer; Foss, Sweden) as described in AOAC method 981.10; 2016. CP was calculated as 6.25 × N. The gross energies (GEs) of feeds, refusals, PA, dried feces, and acidified urine were determined using an automatic isoperibol calorimeter (6400EF, Parr Instrument Company, Moline, IL, USA). FA concentrations in feed and PA were measured by the direct methylation method (32), using previously described analysis techniques (33).

The ammonium nitrogen (NH_3_-N) concentration in rumen fluid obtained from experiment 2 (described below) was determined using a modified colorimetric method (34). For determination of volatile fatty acids (VFAs), a 5.0-ml aliquot of rumen fluid was mixed with 1.0 ml 25% HPO_3_ and 0.2 ml 2% pivalic acid (35); the mixture was analyzed using previously described methods (36).

#### 2.2.4 Calculations

Energy partitioning was determined by subtracting the energy losses in feces, urine, and CH_4_, as well as daily heat production, from the GE intake as detailed in Sinz et al. (15). Heat energy (HE) was calculated with correction for the assumed CO_2_ production from microbial fermentation based on the equation given in Chawlibog et al. (37).

### 2.3 Experiment 2: effect of PA on rumen fermentation and protozoal populations in Holstein steers

In experiment 2, five rumen fistulated Holstein steers with an initial BW of 744.8 ± 15.8 kg (mean ± standard error) were randomly allocated to individual feeders equipped with steel stanchions. All animals were adapted to the same basal diet (Table 1) for 25 days (TMR) then supplemented with PA at 5% DM (TMR-PA) of the basal diet for 38 days. The diet was provided at 2% initial BW twice daily (09:00 and 18:00), and the animals had unrestricted access to water. Approximately 200 ml of ruminal fluid were collected from each animal through the cannula before feeding (0 h), and at 1.5 and 3 h after feeding, on the last day of each period; all fluid was strained through four layers of muslin. Immediately after the pH had been measured with a Seven Easy pH meter (Mettler-Toledo, Schwerzenbach, Switzerland), the ruminal fluid was transferred to a 50-ml centrifuge tube, snap-frozen in liquid N, and stored at −80°C until further analysis.

#### 2.3.1 Quantification of rumen protozoal abundance using RT-qPCR

Rumen liquor collected after 3 h of morning feeding from cannulated Holstein steers was subjected to genomic DNA extraction using previously described methods (36). Real-time PCR assays to determine the relative abundances of major rumen protozoal species such as *Entodinium caudatum, Epidinium caudatum, Eudiplodinium maggii*, and *Polyplastron multivesiculatum* were performed using the SYBR Green Real-Time PCR Master Mix (Bioneer, Daejeon, Korea) and the CFX96 Touch™ Real-Time PCR Detection System with previously described analysis methods and PCR conditions (10). All primers (Table 2) were designed using the Primer-BLAST tool based on National Center for Biotechnology Information (NCBI) published sequences (www.ncbi.nlm.nih.gov, accessed on December 22, 2021). All primers were optimized for the annealing temperature with the highest product band intensity, which was determined by multiple gradient PCR assays. The primers targeted the 18S ribosomal region or other species-specific conserved coding region that lacked potential matches with other nucleotide sequences available in the NCBI. The 2^−Δ*ΔCT*^ method was used to determine relative fold-changes (38), and all data were normalized to the abundance of total protozoa.

**Table 2:** Oligonucleotide primers used for real-time PCR assays.

**S. No**	**Primer name**		**Sequence**	**NCBI accession No**.	**Product size (bp)**
1	*Entodinium caudatum*	FP	ATTCATCCAAGCCTCAAGAGAAGT	AM051654.1	144
		RP	TGTCATTTTCCCATTCGACCTTGA		
2	*Epidinium caudatum*	FP	ACTGAAATGGGAGCCACTGA	AB011273.1	186
		RP	TGGTTCAAAGGTTCCAGCAGA		
3	*Eudiplodinium maggii*	FP	TCAGGCTGCACTGGAAAACA	AM419456.1	185
		RP	CCCAATCCCATCCATTGCCT		
4	*Polyplastron multivesiculatum*	FP	CATTGATGGCAACCCTACATTT	AJ516958.1	114
		RP	AGTCCTTTACTCGTCCATGCT		
5	Total protozoa	FP	TTTGTACACACCGCCCGTC		135
		RP	GGTTCACCTACGGAAACCTTGT		

### 2.4 Statistical analyses

A Shapiro–Wilk test was performed to check the normality of the data. When data were not normally distributed, they were transformed using the PROC RANK procedure of SAS (version 9.4, SAS Institute, Cary, NC, USA). Data were subjected to mixed-model analysis of variance in SAS, considering the treatment and period as fixed effects and animals as the random effect. Pairwise comparisons among least-squares means were performed by the Tukey-Kramer test. The ruminal fermentation characteristics (pH, VFA, and NH_3_) were analyzed by repeated-measures analysis of variance. The Akaike information criterion was used to determine which covariance structure had the best fit; based on the results, an “autoregressive (AR1)” analysis was chosen for assessment of fermentation characteristics. Means were calculated using the LSMEANS function, then compared using the PDIFF option in SAS. Differences were considered statistically significant at *p* < 0.05, and trends were considered at 0.05 ≤ *p* < 0.10.

## 3 Results

### 3.1 Chemical composition of the PA and diet

The proximate, FA composition, and metabolite profile of PA are reported in our previous study (25). Briefly, the analysis of the FA composition of PA revealed enrichment of saturated FAs (SFAs; 3.5 g/kg DM), monounsaturated fatty acids (MUFAs; 2.3 g/kg DM), and PUFAs (5.1 g/kg DM) dominated by linoleic acid (C18:2; 4.5 g/kg DM). Analytes such as caffeic acid, chlorogenate, quercetin, quercetin-3-O-glucoside and dioctyl sulfosuccinate were present in high intensities (25). The actual concentrations of these metabolites are yet to be determined.

The TMR offered to animals in both periods had a similar nutrient composition, except that the EE content of TMR-PA was 8% greater than the EE content of the TMR (Table 1) because of the higher EE concentration in PA (25). This also led to greater polyunsaturated FA and total FA concentrations in the TMR-PA diet (Supplementary Table 1) compared with TMR.

### 3.2 Effects of PA in Hanwoo steers

#### 3.2.1 Intake, digestibility, and performance

There were no significant differences (*p* > 0.05) in nutrient intake across dietary treatments (Table 3), but an increase (*p* < 0.001) in apparent nutrient digestibility was observed with maximum increases of 8.4% and 18.6% in OM and NDF digestibility, respectively in TMR-PA compared with TMR. There were no changes (*p* > 0.05) in ADG or feed conversion ratio in response to PA supplementation.

**Table 3:** Least-squares means of body weight, feed intake, and apparent total tract digestibility of Hanwoo steers (*n* = 6) supplemented with *Pharbitis nil* seeds at 5% DM (TMR-PA).

**Item**	**TMR**	**TMR-PA**	**SEM**	***p*-value**
Initial BW, kg	459.01	480.67	26.12	
Final BW, kg	480.67	521.83	26.87	
Average daily gain, kg/day	1.03	0.91	0.10	0.190
Feed conversion ratio	9.68	9.24	1.33	0.231
**Intake, kg/day**				
Dry matter (DM)	7.72	7.93	0.35	0.169
Organic matter (OM)	7.20	7.40	0.33	0.158
Crude protein (CP)	1.26	1.33	0.06	0.029
Ether extract (EE)	0.37	0.41	0.02	0.001
Neutral detergent fiber (aNDFom)	2.99	3.08	0.14	0.117
Acid detergent fiber (ADFom)	1.43	1.45	0.06	0.289
Gross energy (GE, Mcal/day)	33.74	34.86	1.55	0.103
**Digestibility, %**				
DM	65.13	71.08	0.87	< 0.0001
OM	67.46	73.22	0.82	0.001
CP	69.67	71.89	0.88	0.082
EE	73.49	70.57	1.65	0.255
aNDFom	51.58	63.41	1.21	< 0.0001
ADFom	55.62	61.22	1.41	0.011
GE	67.12	71.97	0.92	0.003

TMR, total mixed ration; TMR-PA, TMR supplemented with Pharbitis nil seeds (5% DM).

FCR, feed conversion ratio (kg DM intake/kg BW gain); aNDFom, neutral detergent fiber assayed with heat-stable amylase and expressed exclusive of residual ash; ADFom, acid detergent fiber expressed excluding residual ash; SEM, standard error of the mean.

#### 3.2.2 Methane emission

A pronounced decrease (*p* = 0.079) in daily absolute CH_4_ production was observed in animals supplemented with PA at 5% DM intake (TMR-PA; Table 4). Relative decreases (*p* < 0.05) of 17.2% and 17.5% were observed when the results were expressed as CH_4_ (g/kg OM) and CH_4_ (g/kg NDF), respectively. The reduction in CH_4_ yield was particularly pronounced (*p* < 0.005), with levels of 24.0% and 33.3% expressed for digestible OM and NDF intakes (Table 4). An 18.1% decrease in GE intake lost as CH_4_ (Ym) was also observed in animals supplemented with PA, relative to animals fed only TMR.

**Table 4:** Effects of *Pharbitis nil* seeds supplemented at 5% DM (TMR-PA) on enteric methane emission from Hanwoo steers (*n* = 6).

**Item**	**TMR**	**TMR-PA**	**SEM**	***p*-value**
**Intake (chamber), kg/day**				
Dry matter (DM)	7.27	7.73	0.29	0.001
Organic matter (OM)	6.78	7.22	0.27	0.001
Neutral detergent fiber (aNDFom)	2.82	3.01	0.11	0.001
Gross energy (GE, Mcal/day)	31.78	34.02	1.27	0.001
**CH**_4_ **emissions**				
CH_4_ (g/day)	165.92	145.05	7.65	0.079
CH_4_ (g/kg DMi)	22.83	18.92	0.78	0.016
CH_4_ (g/kg OMi)	24.46	20.26	0.85	0.015
CH_4_ (g/kg NDFi)	58.92	48.60	2.03	0.014
CH_4_ (g/kg dOMi)	36.31	27.61	1.53	0.004
CH_4_ (g/kg dNDFi)	114.87	76.56	6.46	0.002
Ym	6.92	5.67	0.25	0.014

DMi, dry matter intake; OMi, organic matter intake; NDFi, neutral detergent fiber intake; dOMi, digestible organic matter intake; dNDFi, digestible NDF intake; Ym, CH_4_ energy expressed as %GE intake; SEM, standard error of the mean.

#### 3.2.3 N balance

The supplementation of PA at 5% DM intake (TMR-PA) did not affect (*p* > 0.05) daily fecal or urinary N excretion amount (Table 5). However, when expressed as the proportion of total N intake, a 7.9% and 7.6% decrease (*p* < 0.05) in fecal and urinary N excretion were noted, respectively. Overall, a 7.7% decrease (*p* < 0.05) in total N excretion and 30.3% increase (*p* < 0.05) in N retention in the body were observed in animals fed PA compared with the non-PA-fed group (Table 5).

**Table 5:** Effects of *Pharbitis nil* seeds supplemented at 5% DM (TMR-PA) on nitrogen balance in Hanwoo steers (*n* = 6).

**Item**	**TMR**	**TMR-PA**	**SEM**	***p*-value**
DMI, kg/day	7.72	7.93	0.35	0.169
Feces excreted, kg DM/day	2.69	2.29	0.14	0.010
Urine excreted, kg/day	10.03	9.22	1.08	0.391
Nitrogen (N) intake, g/day	202.01	213.23	9.01	0.026
Fecal N excretion, g/day	61.47	59.62	3.76	0.569
Urinary N excretion, g/day	99.80	97.67	5.78	0.420
Total N excretion, g/day	161.27	157.30	8.32	0.283
Body N retention, g/day	40.74	55.93	2.50	0.003
**% N intake**				
Fecal N	30.32	27.91	0.87	0.054
Urinary N	49.44	45.70	1.61	0.077
Total excreted N	79.75	73.61	1.18	0.003
Retained N	20.25	26.39	1.18	0.003
**% total N excreted**				
Fecal N	38.03	38.05	1.42	0.990
Urinary N	61.98	61.96	1.42	0.990

SEM, standard error of the mean.

#### 3.2.4 Energy balance

No difference (*p* > 0.05) in CO_2_ production was observed between the experimental groups (Table 6). The respiratory quotient was observed to be greater (*p* < 0.05) in TMR fed animals. Although the GE intake did not differ between treatments, the fecal, urine and CH_4_ energy losses decreased (*p* < 0.005) when PA was included in the diet, thereby increasing (*p* < 0.005) the supplies of digestible energy (DE) and metabolizable energy (ME) by 7.2% and 14.8%, respectively, when expressed as % GEI (Table 7). However, only a numerical increase (*p* > 0.05; Table 7) in retained energy (RE) was observed in animals supplemented with PA. The relationship between ME and DE differed (*p* < 0.005) between the treatments; correlation coefficients of 0.81 and 0.87 were obtained for the TMR and TMR-PA groups, respectively. The utilization efficiencies of the ME to RE were not significantly different (*p* > 0.05); they averaged 0.21 and 0.25 for the TMR and TMR-PA groups, respectively (Table 7).

**Table 6:** Effects of *Pharbitis nil* seeds supplemented at 5% DM (TMR-PA) on heat production in Hanwoo steers (*n* = 6).

**Item**	**TMR**	**TMR-PA**	**SEM**	***p*-value**
O_2_ consumption, L/day	3,180.83	3,619.88	249.35	0.033
CO_2_ production, L/day	3,614.35	3,601.73	239.48	0.959
CH_4_ production, L/day	232.17	203.03	15.28	0.079
RQ	1.14	1.00	0.03	0.025
Ratio CH_4_/CO_2_ in breath	0.07	0.06	0.00	0.004
Urinary N, g/day	99.80	97.67	5.78	0.420
HP, Mcal/day	14.45	16.40	1.13	0.049

RQ, respiratory quotient (CO_2_ production/O_2_ consumption); SEM, standard error of the mean; HP = heat production (Mcal/day) corrected for assumed CO_2_ production from microbial fermentation = [0.01618 × O_2_ (L/day) + 0.00502 × (CO_2_ (L/day) – 3 × CH_4_ (L/day)) – 0.00217 × CH_4_ (L/day) – 0.00599 × urinary N (g/day)]/4.184.

**Table 7:** Effects of *Pharbitis nil* seeds supplemented at 5% DM (TMR-PA) on energy balance in Hanwoo steers (*n* = 6).

**Item**	**TMR**	**TMR-PA**	**SEM**	***p*-value**
Gross energy (GE, Mcal/day)	33.74	34.86	1.55	0.103
**Loss, Mcal/day**				
Feces	11.11	9.78	0.63	0.033
Urine	1.98	1.38	0.11	0.004
Methane	2.33	1.98	0.13	0.012
Heat	14.45	16.40	1.13	0.049
Digestible energy (DE), Mcal/day	22.63	25.09	1.04	0.001
Metabolisable energy (ME), Mcal/day	18.32	21.73	1.00	0.001
Retained energy (NE), Mcal/day	3.87	5.34	0.77	0.192
**Loss, %**				
Feces, % GE	32.86	28.01	0.92	0.003
Urine, % GE	5.95	4.02	0.48	0.029
Methane, % GE	6.92	5.68	0.25	0.013
Heat, % GE	42.84	46.74	1.96	0.190
DE, % GE	67.14	71.99	0.92	0.003
ME, % GE	54.28	62.29	1.01	0.001
NE, % GE	11.45	15.56	2.23	0.180
GE, Mcal/kg DM	4.37	4.40	0.00	< 0.0001
DE, Mcal/kg DM	2.94	3.17	0.04	0.002
ME, Mcal/kg DM	2.37	2.74	0.04	0.001
RE, Mcal/kg DM	0.50	0.68	0.10	0.172
ME/DE	0.81	0.87	0.01	0.003
RE/ME	0.21	0.25	0.04	0.403

SEM, standard error of the mean.

### 3.3 Effects of PA on fermentation parameters and protozoal populations in Holstein steers

**Figure 1:**
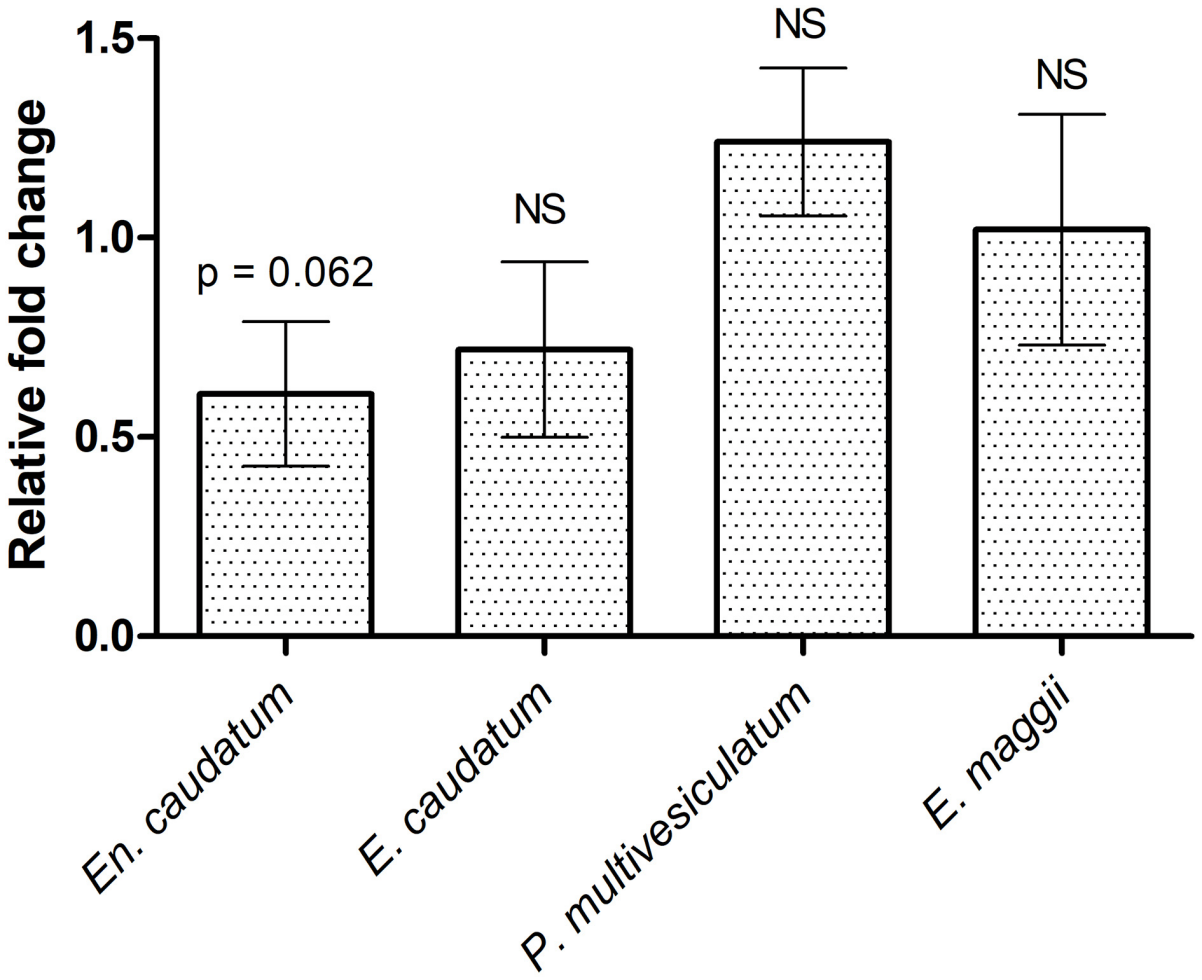
Effect of supplementing *Pharbitis nil* seeds at 5% DM intake on rumen protozoal abundance in cannulated Holstein steers (*n* = 5).

The average ruminal pH and NH_3_-N concentration decreased (*p* < 0.05) in cannulated Holstein steers supplemented with PA (Table 8). The NH_3_-N concentration was 56.3% lower in the TMR-PA group than in the TMR group after 1.5h feeding; it continued to remain lower after 3 h feeding. An increase (*p* = 0.087) in the total VFA concentration after 3 h of feeding, accompanied by a 26.6% increase (*p* < 0.05) in the propionate proportion, was also observed in the TMR-PA group. This clearly led to a decrease (*p* < 0.005) in the acetate/propionate ratio. Analysis of rumen protozoal populations revealed a 40% reduction (*p* = 0.062) in the relative abundance of the protozoal species *En. caudatum* among animals supplemented with PA (Figure 1). There were no significant differences in the abundances of other protozoal species.

**Table 8:** Ruminal fermentation characteristics in cannulated Holstein steers supplemented with *Pharbitis nil* seeds at 5% DM (*n* = 5).

**Item**	**TMR**			**TMR-PA**			**SEM**	***p*** **-value**		
**Time interval**	**0 h**	**1.5 h**	**3 h**	**0 h**	**1.5 h**	**3 h**		**Treatment**	**Hours**	**Treatment** × **hours**
pH	7.17	6.44	6.55	6.81	6.30	6.27	0.09	0.001	0.117	0.006
Total VFA, mM	63.54	121.13	104.46	72.01	121.32	118.10	6.14	0.087	< 0.0001	0.419
VFA, %
Acetate	66.50	57.86	60.75	64.78	54.93	56.24	1.05	0.019	< 0.0001	0.136
Propionate	17.83	26.15	22.15	19.73	29.26	28.05	0.94	0.002	< 0.0001	0.025
Iso-butyrate	1.89	1.28	1.34	1.83	1.27	1.24	0.07	0.184	< 0.0001	0.820
Butyrate	9.30	10.47	11.35	8.90	9.72	9.85	0.36	0.060	< 0.0001	0.067
Iso-valerate	3.10	2.19	2.32	3.29	2.64	2.33	0.21	0.303	< 0.0001	0.290
Valerate	1.38	2.05	2.10	1.48	2.18	2.30	0.11	0.136	< 0.0001	0.756
Acetate:Propionate	3.75	2.27	2.78	3.30	1.88	2.01	0.16	0.004	< 0.0001	0.191
NH_3_-N, mg/dl	10.98	25.38	17.43	5.49	11.09	11.89	1.48	< 0.0001	< 0.0001	0.006

SEM, standard error of the mean.

## 4 Discussion

Dietary strategies to mitigate CH_4_ and N emissions from ruminants must be commercially practical, without negative impacts on digestibility and animal performance. In our previous *in vitro* evaluations (10) we demonstrated the anti-methanogenic potential of *P. nil* seeds that are widely distributed in East Asian countries. We also observed increases in the *in vitro* DM and NDF digestibility with increasing supplementation of PA; the maximum response was recorded at 9% DM supplementation (25). A consistent response was present in the current *in vivo* trial at a supplemental level of 5% DM; the magnitude of increase in digestibility was comparable with our previous *in vitro* observation (25). This increased digestibility led to increased DE intake for animals in the treatment period compared with the control period, although there were no differences regarding GE intake. The increase in nutrient digestibility may be partially explained by the laxative effect of PA, which improved intestinal motility (24). There is evidence that altered intestinal motility and contractions create turbulence and convective currents that transport nutrients from the center of the lumen toward the epithelium, eventually improving nutrient absorption (39). Furthermore, dioctyl sulfosuccinate, an anionic surfactant substance traditionally recommended as a laxative and stool softener, was reported to be present in PA in our previous study (25). Another possible explanation is that better synchronization between carbohydrates and N occurred when less ammonia was available to rumen microbes in the treatment period, compared with the control period. This might have increased the growth of cellulolytic bacteria and enhanced fiber digestibility (40). The observed increase and decrease in rumen total VFA and pH in treatment period, respectively, provide further support to this hypothesis. Increases in fibrolytic bacterial populations were also observed in our previous study (25) when PA was incubated in the rumen of Holstein steers. Although the energy balance trial and rumen fermentation trial were performed in two different animal breeds, the basal diet fed was same in the both trials and hence relating their effects can be meaningful.

Greater NDF digestion stimulates acetate fermentation, which may enhance CH_4_ and CO_2_ emission (41). In this study, an inverse effect was observed that favored propionate production and decreased CH_4_ yield, consistent with our previous study (25). This effect led to a decrease in CH_4_ energy loss for animals in the treatment period, compared with the control period. Furthermore, the observed CH_4_ yield and Ym from Hanwoo steers fed TMR in the experiment were comparable with previously reported values (42). The proportion of CH_4_ energy loss to DE intake in the present study (10.3%) was comparable with values from growing Hanwoo steers fed different diets, as reported in our previous study (43). However, the magnitude of CH_4_ reduction via PA supplementation in the present study was 2.5-fold greater than the magnitude observed in our previous *in vitro* study with a similar level of PA supplementation (25). There are several possible reasons for this, including differences in culture system (44), animal breed (42), and diet. Furthermore, CH_4_ reduction in the treatment period may be partially explained by the greater polyunsaturated FA concentration, including dietary linoleic acid (C18:2n6c) from the supplemented PA (25). The role of the dietary FA concentration in ruminal CH_4_ production is well-known (45). Beauchemin et al. (46) reported that a decrease in CH_4_ yield (g/kg DM intake) was accompanied by a decrease in the number of rumen protozoa when cows were fed crushed linseed, similar to our results. Defaunation has been associated with lower levels of methanogenesis (26) due to the symbiotic relationship between methanogens and protozoa (47). Dohme et al. (48) also reported the antiprotozoal actions of FAs, including C18:2n6c, which produce considerable decreases in CH_4_ production. However, in the present study, supplementation with PA alone increased the added FA concentration by 0.4%, which was insufficient to achieve the observed reductions in protozoal population and CH_4_ (45). This result suggests that other metabolites in PA (e.g., quercetin and quercetin-3-O-glucoside) exhibit antiprotozoal action, as demonstrated in previous studies (49, 50). In our previous studies, we observed a moderate binding of these metabolites to the *cyclic guanosine monophosphate-dependent protein kinase* of *En. Caudatum* (25), and also a drastic decrease in total protozoal population in PA treated *in vitro* rumen buffer (10), further supporting our results. However, the concentrations of these metabolites in PA have not been quantified. Furthermore, *Entodinium* spp. reportedly constitute only 10–15% of the total protozoal population; they are dominated by *Ophryoscolex* spp. or other unclassified families (51, 52). Therefore, the decrease in *En. caudatum* abundance alone could not completely explain the decrease in CH_4_ production observed in this study. Another possible factor may be the laxative effect of PA, which could have increased the rate of feed passage and requires further investigation. The elimination of rumen protozoa is consistently associated with an increase in the propionate proportion (2), which matches the observed 26.6% increase in propionate proportion during the treatment period in Holstein steers. This finding suggested that propionate synthesis was the major H_2_ sink, thereby reducing H_2_ available for CH_4_ synthesis in animals supplemented with PA (41). Furthermore, defaunation has been related to a decrease in nutrient digestibility (26); such a decrease was not observed in the present study. This was possibly related to the lack of change in the abundances of cellulolytic genera such as *Epidinium, Polyplastron*, and *Eudiplodinium*, and a decrease in the abundance of *Entodinium*, which is weakly hemicellulolytic (53).

The increases in ruminal protein degradation and peptide deamination cause increased production of NH_3_-N, which is absorbed through the rumen wall and excreted as urea in urine, leading to N losses in a highly volatile form (54). The 56% decrease in the rumen NH_3_-N concentration in cannulated Holstein steers supplemented with PA could be related to the decreased urinary N excretion and increased N retention in Hanwoo steers in the current experiment. These findings suggest that the ruminal degradability of dietary CP is reduced, which may be related to the overall decrease in protozoa observed in our current and previous studies (25). However, there was no negative effect on the total tract digestibility of CP, which suggested hind gut digestion and CP utilization. Additionally, consistent with the present study, defaunation is associated with lower NH_3_-N levels in rumen fluid (2), due to a decrease in the microbial grazing of *En. caudatum* (55), which deaminates the engulfed microbial protein to NH_3_-N (26). Previous studies (49, 50) have also shown decreases in protozoal ammoniagenesis after the addition of plant extracts that (like PA) are rich in quercetin and quercetin-3-O-glucoside. The minimum required NH_3_-N concentration for maximum microbial growth in the rumen is 5 mg/dl (56). A previous study showed that levels of 5–8 mg/dl were sufficient to favor fiber digestion, whereas levels of >8 mg/dl did not increase feed degradation in the rumen (57). The NH_3_-N concentration of 5.5–11.9 mg/dl upon supplementation with PA would therefore meet the minimum requirement for microbial protein synthesis. However, studies on the rumen microbial protein synthesis should be carried out to further support the increased N utilization noted in PA fed animals. The proportion of N retained from N intake for Hanwoo steers fed only a basal diet (18%−20%) observed in this study was bit greater than proportions (16%−18%) observed in other breeds of beef cattle (13, 58, 59).

The decrease in urinary N excretion in animals supplemented with PA also led to a decrease in the urinary energy (UE) content (from 6% GE intake to 4% GE intake) because UE is closely associated with UN due to the higher energy contents of urinary hippuric acids, amino acids, and creatinine (60). The ratio of UE losses to the GE and DE intakes (8.8%) in the present study was much higher than the ratio among Hanwoo steers in the early fattening stage, reported in our previous work (43, 61). This discrepancy may be related to the greater dietary CP content of 16.6% in the present study, compared with the value of 12.5% in the previous studies, which might have increased the UN excretion while enhancing UE loss.

The decreased ratio of energy intake to energy excretion in feces, urine, and enteric CH_4_ resulted in greater ME intake in animals supplemented with PA compared with animals fed only TMR. The observed DE intake and ME intake for Hanwoo steers in the present study were comparable with the results in our previous work that consolidated many studies involving Hanwoo steers with different BWs and diets (43). Additionally, the DE intake and ME intake values were within the ranges of values reported for other beef cattle breeds (1.8–3.8 and 1.4–3.5 Mcal/kg of DM, respectively) (62). The observed efficiency of converting DE to ME in animals fed a basal diet in the present study (0.81) was similar to the efficiencies recommended by the Agricultural Research Council (63), National Research Council (64), and Commonwealth Scientific and Industrial Research Organization (65). However, it was below the efficiencies within the British feeding system (66) and other recent studies (43, 62, 67), which ranged from 0.86 to 0.90 for several beef cattle breeds (Hanwoo, Friesian, Hereford, Brahman, Thai, Angus, Hereford-Angus, Jersey, MARC II composite, Holstein, Charolais-cross, and Continental-British). Similarly, the utilization efficiency of ME (RE:ME) was 0.21 under 2.4 Mcal/kg DM of ME intake, which was lower than the reported value of 0.29 considering feeds with ME intakes of 2.0 Mcal/kg DM (64). However, this discrepancy may be due to differences in animal breeds and diets between experiments. Moreover, the ME:DE (0.87) and RE:ME (0.25) ratios for animals supplemented with PA were similar to the ratios for suggested values (43, 62, 67), presumably due to increases in DE intake and ME intake upon supplementation with PA.

Increases in ruminant productivity (e.g. ADG) via reduction of enteric CH_4_ emissions and enhancement of energy conversion efficiency is the desired outcome of a CH_4_ mitigation strategy. Similarly, Li and Guan (68) reported that increases in N metabolism and utilization contributed to increased feed efficiency in beef cattle. However, decreases in CH_4_, N emission, and other energy losses in the treatment period did not significantly contribute to the increase in RE, may be due to the increased heat energy loss. This result might also be due to the digestion of metabolites such as phenols and tannins (chlorogenate in high intensities) in PA (25), which may require detoxification with the cost of additional energy (69). Furthermore, CH_4_ mitigation and urinary N mitigation in the treatment period were insufficient for reduction of the calculated HE because small coefficients for CH_4_ and urinary N were used in the equation for HE estimation. As a result, RE only increased by 1.3 Mcal/day in the treatment period; which eventually did not contribute much to changes in the ADG and feed conversion efficiency. There were also individual animal variations in heat production that contributed to the lack of statistical significance for RE, suggesting that future studies should be conducted over a longer period with additional animal replicates.

## 5 Conclusions

Seeds of *P. nil* demonstrated promise for mitigating CH_4_ emissions from *in vitro* rumen fermentation studies. They were very efficient in live animals, leading to a 17.2% decrease in CH_4_ yield (g/kg OM) when supplemented at 5% DM. The supplemented feed was also palatable, demonstrated high nutritional value and a 7.6% decrease in urinary N loss, and exhibited a >10% increase in nutrient digestibility. Metabolizability (ME/GE) and ME utilization (RE/ME) were also improved, suggesting that PA was a valid alternative to ionophores or other growth promoters. Greater retention of dietary N and E may contribute to improvements in production parameters, thereby reducing age at slaughter, with environmental and economic benefits. However, the increase in heat production was a notable limitation. Future studies should focus on profiling rumen and blood metabolites to identify any potential PA-induced toxicity related to the increased HE. The rumen microbial metagenome should also be evaluated to better understand the beneficial effects of PA on digestibility and energy conversion efficiency.

## Data Availability

The original contributions presented in the study are included in the article/Supplementary material, further inquiries can be directed to the corresponding authors.
