# Supplementary material for: Methane emission, nitrogen excretion, and energy partitioning in Hanwoo steers fed a typical TMR diet supplemented with Pharbitis nil seeds
Source: Front Vet Sci. 2024 Sep 24;11:1467077. doi: 10.3389/fvets.2024.1467077 (PMC11459670; doi:10.3389/fvets.2024.1467077)
Supplement: Supplementary file 1 [file Table_1.docx]

Table S1. Fatty acid composition of the dietary treatments

| **Fatty acid (mg/100 DM)** | **TMR** | **TMR-PA** |
| --- | --- | --- |
| Caprylic acid (C8 :0) | 1.29 | 1.45 |
| Capric acid (C10 :0) | 3.45 | 3.88 |
| Lauric acid (C12 :0) | 65.99 | 62.85 |
| Myristic acid (C14 :0) | 31.01 | 30.67 |
| Pentadecylic acid (C15 :0) | 1.77 | 1.88 |
| Palmitic acid (C16 :0) | 618.38 | 698.86 |
| Palmitoleic acid (C16 :1) | 9.54 | 11.21 |
| Margaric acid (C17 :0) | 4.21 | 4.5 |
| Stearic acid (C18 :0) | 107.65 | 140.42 |
| Elaidic acid (trans 9 C18 :1) | 1.42 | 2.81 |
| Oleic acid (cis 9 C18 :1) | 948.16 | 1010.29 |
| Trans-linoleic acid (C18 :2n6t) | 0.11 | 1.46 |
| Linoleic acid (C18 :2n6c) | 1875.05 | 2000.68 |
| Gamma-Linolenic acid (C18 :3n6) | 0 | 2.9 |
| Alpha linolenic acid (C18 :3n3) | 99.91 | 116.74 |
| Arachidic acid (C20 :0) | 17.12 | 23.33 |
| Gondoic acid (C20 :1n9) | 12.41 | 12.16 |
| Eicosadienoic acid (C20 :2n6) | 1.87 | 2.17 |
| Arachidonic acid (C20 :4n6) | 13.46 | 13.3 |
| Heneicosylic acid (C21 :0) | 1.61 | 1.82 |
| Behenic acid (C22 :0) | 6.03 | 9.84 |
| Erucic acid (C22 :1n9) | 0.57 | 0.55 |
| Tricosylic acid (C23 :0) | 0 | 0.11 |
| Lignoceric acid (C24:0) | 9.84 | 11.81 |
| Adrenic acid (C24 :1n9) | 0.61 | 0.58 |
| SFA | 867.06 | 989.99 |
| MUFA | 972.71 | 1037.64 |
| MUFA:SFA | 1.12 | 1.05 |
| Omega-6 | 1890.49 | 2020.51 |
| Omega-3 | 99.91 | 116.74 |
| Omega6:3 | 18.92 | 17.31 |
| PUFA | 1990.4 | 2137.25 |
| PUFA:SFA | 2.3 | 2.16 |
| Trans fat | 1.53 | 4.28 |
| Total fatty acids (mg/100 g DM) | 3831.46 | 4166.33 |

SFA, saturated fatty acids; MUFA, mono unsaturated fatty acids; PUFA, poly unsaturated fatty acids; SFA = C10:0 + C11:0 + C12:0 + C14:0 + C15:0 + C16:0 + C17:0 + C18:0 + C20:0 + C21:0 + C22:0 + C24:0

MUFA = C14:1n5 + C16:1n7 + C17:1n7 + C18:1n7 + C18:1n9 + C20:1n9 + C22:1n9 + C24:1n9

Omega-6 = C18:2n6 + C18:3n6 + C20:2n6 + C20:3n6 + C20:4n6 + C22:2n6 + C22:4n6

Omega-3 = C18:3n3 + C22:6n3

PUFA = C18:2n6 + C18:2c9,t11 + C18:3n3 + C18:3n6 + C20:2n6 + C20:3n3 + C20:3n6 + C20:4n6 +  C20:5n3 + C22:2n6 + C22:4n6 + C22:5n3 + C22:6n3

Trans fatty acids = C18:1n7t11 + C18:1n9t + C18:2n6t + C18:2c9, t11
